# Clinical practices related to liberation from mechanical ventilation in Latin American pediatric intensive care units: survey of the *Sociedad Latino-Americana de Cuidados Intensivos Pediátricos* Mechanical Ventilation Liberation Group

**DOI:** 10.62675/2965-2774.20240066-en

**Published:** 2024-09-05

**Authors:** Alejandra Retta, Analía Fernández, Ezequiel Monteverde, Cintia Johnston, Andrés Castillo-Moya, Silvio Torres, Jesus Dominguez-Rojas, Matias G. Herrera, Vlademir Aguilera-Avendaño, Yúrika López-Alarcón, Davi Pascual Rojas Flores, Manuel Eduardo Munaico-Abanto, Júlia Acuña, Rosa León, Carla Ferreira, Gabriela Sequeira, Cristina Camilo, Mauricio Yunge, Yolanda López Fernández

**Affiliations:** 1 Hospital General de Niños Ricardo Gutiérrez Intensive Care Unit Buenos Aires Argentina Intensive Care Unit, Hospital General de Niños Ricardo Gutiérrez - Buenos Aires, Argentina.; 2 Hospital General de Agudos Carlos G. Durand Intensive Care Unit Buenos Aires Argentina Intensive Care Unit, Hospital General de Agudos Carlos G. Durand - Buenos Aires, Argentina.; 3 Universidade Federal de São Paulo Department of Pediatrics São Paulo SP Brazil Department of Pediatrics, Universidade Federal de São Paulo - São Paulo (SP, Brazil; 4 Pontificia Universidad Católica de Chile Intensive Care Unit Santiago Chile Intensive Care Unit, Pontificia Universidad Católica de Chile - Santiago, Chile; 5 Hospital Universitario Austral Pilar Argentina Hospital Universitario Austral- Pilar, Argentina; 6 National Hospital Edgardo Rebagliati Martins Department of Pediatrics Lima Peru Department of Pediatrics, National Hospital Edgardo Rebagliati Martins - Lima, Peru.; 7 Hospital de Pediatría Prof. Dr. Juan P. Garrahan Intensive Care Unit Buenos Aires Argentina Intensive Care Unit, Hospital de Pediatría Prof. Dr. Juan P. Garrahan - Buenos Aires, Argentina; 8 Hospital del Niño Dr. Ovidio Aliaga Uría Intensive Care Unit La Paz Bolivia Intensive Care Unit, Hospital del Niño Dr. Ovidio Aliaga Uría - La Paz, Bolivia.; 9 Hospital General de Medellín Luz Castro de Gutiérrez Intensive Care Unit Medellin Colombia Intensive Care Unit, Hospital General de Medellín Luz Castro de Gutiérrez - Medellin, Colombia; 10 Instituto Mexicano del Seguro Social Hospital General Regional nº 1 Intensive Care Unit Chihuahua Mexico Intensive Care Unit, Hospital General Regional nº 1, Instituto Mexicano del Seguro Social - Chihuahua, Mexico.; 11 National Hospital Edgardo Rebagliati Martins Intensive Care Unit Lima Peru Intensive Care Unit, National Hospital Edgardo Rebagliati Martins - Lima, Peru.; 12 Instituto de Medicina Tropical Intensive Care Unit Asunción Paraguay Intensive Care Unit, Instituto de Medicina Tropical - Asunción, Paraguay.; 13 Instituto Nacional de Salud del Niño Lima Peru Instituto Nacional de Salud del Niño - Lima, Peru.; 14 Hospital Universitario San Lorenzo Intensive Care Unit Asunción Paraguay Intensive Care Unit, Hospital Universitario San Lorenzo - Asunción Paraguay.; 15 Centro Hospitalario Pereira Rossell Montevideo Uruguay Centro Hospitalario Pereira Rossell - Montevideo, Uruguay; 16 Hospital de Santa Maria Lisboa Portugal Hospital de Santa Maria - Lisboa, Portugal.; 17 Clínica Las Condes Intensive Care Unit Las Condes Chile Intensive Care Unit, Clínica Las Condes - Las Condes, Chile.; 18 Hospital Universitario Cruces Intensive Care Unit Barakaldo Spain Intensive Care Unit, Hospital Universitario Cruces - Barakaldo, Spain.

**Keywords:** Respiration, artificial, Intensive care units, pediatric, Airway extubation, Noninvasive ventilation, Surveys and questionnaires

## Abstract

**Objective:**

To address the current practice of liberating patients from invasive mechanical ventilation in pediatric intensive care units, with a focus on the use of standardized protocols, criteria, parameters, and indications for noninvasive respiratory support postextubation.

**Methods:**

Electronic research was carried out from November 2021 to May 2022 in Ibero-American pediatric intensive care units. Physicians and respiratory therapists participated, with a single representative for each pediatric intensive care unit included. There were no interventions.

**Results:**

The response rate was 48.9% (138/282), representing 10 Ibero-American countries. Written invasive mechanical ventilation liberation protocols were available in only 34.1% (47/138) of the pediatric intensive care units, and their use was associated with the presence of respiratory therapists (OR 3.85; 95%CI 1.79 - 8.33; p = 0.0008). The most common method of liberation involved a gradual reduction in ventilatory support plus a spontaneous breathing trial (47.1%). The mean spontaneous breathing trial duration was 60 - 120 minutes in 64.8% of the responses. The presence of a respiratory therapist in the pediatric intensive care unit was the only variable associated with the use of a spontaneous breathing trial as the primary method of liberation from invasive mechanical ventilation (OR 5.1; 95%CI 2.1 - 12.5). Noninvasive respiratory support protocols were not frequently used postextubation (40.4%). Nearly half of the respondents (43.5%) reported a preference for using bilevel positive airway pressure as the mode of noninvasive ventilation postextubation.

**Conclusion:**

A high proportion of Ibero-American pediatric intensive care units lack liberation protocols. Our study highlights substantial variability in extubation readiness practices, underscoring the need for standardization in this process. However, the presence of a respiratory therapist was associated with increased adherence to guidelines.

## INTRODUCTION

Between 35.7 and 55% of patients admitted to pediatric intensive care units (ICUs) receive invasive mechanical ventilation (MV).^( 1 - 4 )^ Even in the context of various critical pathological processes, early liberation from invasive respiratory support remains a priority objective to avoid risks, complications, and increased health costs associated with prolonged invasive MV.^( 5 - 8 )^

Despite the broad consensus on minimizing invasive MV duration, the scarcity of evidence on pediatric liberation methods contributes to significant variability in practice.^( 9 - 11 )^ The first International Clinical Practice Guidelines for Pediatric Ventilator Liberation in Pediatrics were recently published. Three core recommendations emphasize the use of standardized protocols to identify patients who meet the criteria for liberation from the ventilator and the application of a bundle of measures to confirm readiness for extubation. These measures should include a spontaneous breathing trial (SBT).^( 12 )^

Although the implementation of these guidelines may contribute to standardizing liberation from MV in children, they have only recently begun to be disseminated, and previous studies have revealed substantial variability in care practices.^( 1 - 5 , 7 - 11 )^

We designed and conducted a survey with the general objective of examining current clinical practices related to liberation from invasive MV in Ibero-American pediatric ICUs. The specific objectives included the following: identify the use of standardized protocols; analyze the criteria and parameters used during liberation from invasive MV; and evaluate the use of noninvasive ventilation (NIV) postextubation. The survey results enabled us to identify areas for improvement and provide support for the development of future studies that contribute to generating evidence in this important area of intensive care medicine.

## METHODS

### Design and study population

The survey design was based on a previously published original document^( 11 )^ that was translated into Spanish, which was subsequently adapted by the Mechanical Ventilation Liberation Group of the Respiratory Committee of the *Sociedad Latino-Americana de Cuidados Intensivos Pediátricos* (SLACIP). The survey, initially published in Spanish and later translated into Portuguese in autumn 2021, was conducted through virtual meetings and distributed to all Ibero-American countries via email addresses provided by national coordinators affiliated with the Mechanical Ventilation Liberation Group. Each email included an invitation letter, and a reminder email was sent 2 - 4 weeks later. Upon agreeing to participate, a representative from each pediatric ICU completed the questionnaire, specifying their profession and role. The Google Form® platform (Google LLC, Mountain View, CA) was used, which required participants to enter a preassigned entry code.

### Survey development

The survey comprised 25 questions related to the process of liberation from MV (Appendix 1 - Supplementary Material) and collected the following: demographic characteristics of the participating pediatric ICUs, including the type of health system, type of hospital and pediatric ICU and number and age range of patients admitted in 2019 (pre-coronavirus disease 2019 [COVID-19] pandemic); information on human resources, including the presence of a respiratory therapist (RT); information on clinical practice, including the definition of the process, the use of a standardized protocol, the use of an extubation readiness test (ERT) or bundle of measures to assess the eligibility of a patient to be liberated from invasive MV, including the SBT type and duration (Appendix 2 - Supplementary Material), the use of other tests, such as the air-leak test, the use of corticosteroids preextubation, the success/failure criteria for liberation, and the timeframe for conducting a new SBT in case of failure; and information on postextubation respiratory support.

All the surveys were completed between November 2, 2021, and May 12, 2022. Surveys lacking demographic data and those identified as high risk for duplicate responses were excluded. Duplications were determined by comparing identical responses regarding the profession of the person responsible for answering the survey, type of hospital, number of annual discharges from each unit and financier of the health care center. Exemption from informed consent was obtained by the Institutional Review Committee of the *Hospital Universitario Austral* in Buenos Aires (Argentina). This study complied with the ethical standards of the committee responsible for human experimentation, as well as the 1975 Helsinki Declaration, along with its most recent amendments.

### Results and operational definitions

The primary outcome was the analysis of current practices related to liberation from MV in children within the Ibero-American critical care setting. This analysis focused on describing the characteristics of the participating pediatric ICUs, availability of human resources, implementation of the liberation process, and postextubation respiratory assistance. Specific details regarding the operational definitions can be found in Appendix 2 (Supplementary Material).

### Statistical analysis

A univariate descriptive analysis was conducted. Categorical variables are presented as the absolute quantity (n) and percentage, whereas numerical variables are presented as the mean and standard deviation (SD) or median and interquartile range (IQR), as appropriate for the observed distribution. For comparisons of categorical results, either the chi-square test or Fisher’s exact test was used. For numerical variables, medians were compared via Kruskal-Wallis analysis of variance. A p value less than 0.05 indicated statistical significance. All analyses were performed via R (version 4.2.1; 2018 The R Foundation for Statistical Computing Platform).

## RESULTS

**Figure 1:**
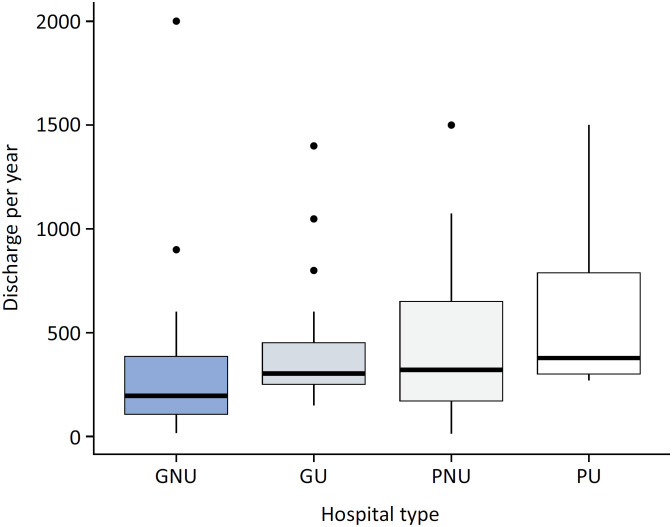
Discharges per year according to the type of hospital.

Invitations to participate were sent to 282 pediatric ICUs, and 138 completed surveys were received (response rate of 48.9%). Among the surveys, 91.3% (126/138) were completed by physicians, and 8.7% (12/138) were completed by RTs. Department heads, directors, or coordinators of pediatric ICUs completed 49.3% (68/138) of the surveys. Most of the responders were affiliated with public hospitals (73.9%, 102/38), and pediatric hospitals accounted for almost a third of them (31.2%, 43/138), with 76.8% (106/138) not being affiliated with a university. Among university-affiliated hospitals, 28.1% (9/32) were general hospitals. The pediatric ICU discharge rate was 300 patients/year (IQ 142 - 432, IQR) ( Figure 1 and Table 1 ).

GNU - general nonuniversity; GU - general university; PNU - pediatric nonuniversity; PU - pediatric university.

**Table 1:** Summary of key survey questions and response rates

Survey questions		Responses %
Profession of respondent (n = 138)		
	Physician	126/138 (91.7)
	Respiratory therapist	30/138 (8.3)
Health system to which the institution belongs (n = 138)		
	Public	102/138 (73.9)
	Private	30/138 (21.7)
	Oher type	6/138 (4.3)
Type of institution * (n = 138)		
	University	32/138 (23.2)
	General	65/138 (47.1)
	Pediatric	43/138 (31.2)
	Other	7/138 (5.1)
Hospital classification by university affiliation (n = 32)		
	General university	9/32 (28.1)
	Pediatric university	23/32 (71.9)
Type of pediatric ICU (n = 138)		
	Medical-surgical	95/138 (68.8)
	Medical-surgical-cardiovascular	35/138 (25.4)
	Other	8/138 (5.7)
Respiratory therapist availability (n = 138)		
	24 × 7	61/138 (44.2)
	Partial (< 12 hours/day)	58/138 (42)
	Not available	19/138 (13.8)
Method used to assess likelihood of extubation (n = 138)		
	SBT only *	37/138 (26.8)
	PS	26/37 (76.5)
	CPAP	2/37 (5.9)
	T-tube	13/37 (38.2)
	GRVS only	36/138 (26.1)
	Both (SBT + GRVS)	65/138 (47.1)
Written protocol (n = 138)		
	Yes	47/138 (34.1)
	No	91/138 (65.9)
ERT components * (n = 47)		
	Daily screening	35/47 (74.5)
	Sedoanalgesia protocol	31/47 (66)
	Criteria for defining ERT failure	31/47 (66)
	SBT	27/47 (57,4)
	Use of preplanned postextubation NIV/CPAP	19/47 (40.4)
	Failure criteria checklist	15/47 (31.9)
SBT length * (n = 125)		
	< 30 minutes	3/125 (2.4)
	30 minutes	38/125 (30.4)
	60 - 120 minutes	81/125 (64.8)
	> 120 minutes	27/125 (21.6)
	Other duration	12/125 (9.6)
Use of an ETT with a cuff (n = 138)		
	Yes	112/138 (81.2)
	No	6/138 (4.3)
	Sometimes	20/138 (14.5)
Protocol-based ETT cuff pressure monitoring (n = 112)		
	Yes	83/112 (74.1)
	No	29/112 (25.9)
Corticosteroids to prevent UAO (n = 125)		
	All patients	49/125 (39.2)
	Patients at high risk of UAO	70/125 (56)
	No indication	3/125 (2.4)
	Unknown	3/125 (2.4)

ICU - intensive care unit; SBT - spontaneous breathing trial; PS - pressure support; CPAP - continuous positive airway pressure; GRVS - gradual reduction of ventilatory support; ERT - extubation readiness testing; NIV - noninvasive ventilation, ETT - endotracheal tube; UAO - upper airway obstruction.

* Multiple responses were allowed.

In terms of pediatric ICU characteristics, 68.8% (95/138) were identified as medical-surgical ICUs, and 25.4% (35/138) were identified as medical-surgical-cardiovascular ICUs. The youngest age at admission was 30 days of age in 64.5% (89/138) of the units, whereas in 29.7% (41/138) of the pediatric ICUs, the minimum age was 7 days. The oldest age at admission was 18 years in 47.1% (65/138), 16 years in 24.7% (34/138) and 14 years in 28.3% (39/138) of the pediatric ICUs.

With respect to the availability of RTs in pediatric ICUs, 44.2% (61/138) reported 24-hour availability, 7.2% (10/138) reported 12-hour availability, and 10.9% (15/138) reported 8-hour availability. In 23.9% (33/138) of the pediatric ICUs, RTs were only accessible for interconsultation, whereas 14% (19/138) of the pediatric ICUs did not have any RT availability.

### Protocols for liberation from invasive mechanical ventilation: extubation readiness testing

Only 47 of the included pediatric ICUs (34.1%, 47/138) reported having a written protocol for liberation from invasive MV. The elements included in the ERT were a sedation and analgesia protocol (66%), criteria for defining ERT failure (66%), a standardized spontaneous ventilation test (57.4%), preestablished criteria for NIV or high-flow nasal cannula (HFNC) postextubation support (40.4%) and an extubation failure checklist (31.9%).

Other measures included in the ERT were the presence of a cough reflex (83.1%), swallowing (41.5%) and muscle strength (26.2%). A total of 66.9% of the professionals reported performing leak tests as part of the ERT via endotracheal tubes (ETTs) with deflated cuffs. With respect to the utilization of cuffed ETTs, a necessary condition for conducting the leak test, the prevalence was greater (91.5%) in units with an in-service RT than in those without (70.1%) an in-service RT (p = 0.0027).

In terms of corticosteroid usage, 56% of the professionals reported prescribing corticosteroids exclusively for high-risk patients prone to airway obstruction, whereas 39.2% reported prescribing corticosteroids exclusively for all patients. Additionally, 42.4% reported the routine administration of nebulized epinephrine.

Extubation readiness testing in pediatric ICUs was significantly associated with the presence of an in-service RT and MV liberation protocols (p = 0.0008; odds ratio [OR] 3.85; 95%CI 1.79 - 8.33). This association was not affected by the type of hospital (OR 3.70; 95%CI 1.72 - 8.33) or the type of pediatric ICU (OR 4.00; 95%CI 1.85 - 8.33).

### Methods for evaluating the ability to ventilate spontaneously

The most common method for assessing extubation readiness was the gradual reduction of ventilatory support (GRVS) with an SBT (47.1%). An SBT alone was reported for 26.8% of the ICUs, and the GRVS alone was reported for 26.1%. The respondents could select more than one option for the method and duration. Among those who reported only SBTs, 47.1% used pressure support (PS) over positive end-expiratory pressure (PEEP), 38.2% used a T-piece, 29.4% used PS according to the diameter of the ETT, and 5.9% used continuous positive airway pressure (CPAP). Among the units that preferred the GRVS, 48.6% favored a gradual reduction in PS, 42.9% preferred a gradual reduction in the respiratory rate while on synchronized intermittent mandatory ventilation (SIMV), and 40% opted for volume support ventilation (VSV) ( Table 2 ).

**Table 2:** Methods for evaluating spontaneous ventilation ability

Methods		n (%)	IC95%
Evaluation of the ability to breathe spontaneously * (n = 138)			
	GRVS + SBT	65 (47.1)	38.6 - 55.8
	SBT	37 (26.8)	19.6 - 35.0
	GRVS	36 (26.1)	19.0 - 34.2
Methods for SBT † (n = 37)			
	PS above PEEP	16 (43.2)	27.1 - 60.5
	T-piece	13 (27.0)	20.2 - 52.5
	PS according to the ETT diameter	10 (27.0)	13.8 - 44.2
	CPAP	2 (5.4)	0.7 - 18.2
Methods for the GRVS † # n = 35 ‡			
	Gradual reduction of PS	17 (48.6)	30.4 - 64.5
	Respiratory rate gradual reduction while on SIMV	15 (42.9)	25.5 - 59.2
	VSV	14 (40.0)	23.1 - 56.5
	Others	1 (2.8)	0.7 - 14.5

IC95% - 95% confidence interval; GRVS - gradual reduction in ventilatory support; SBT - spontaneous breathing trial; PS - pressure support; ETT - endotracheal tube; PEEP - positive end-expiratory pressure; CPAP - continuous positive airway pressure; SIMV - synchronized intermittent mandatory ventilation; VSV - volume support ventilation.

* Total sample size;

† multiple responses were allowed;

‡ the sample size was 36, but one response was missing.

The reported duration of SBTs was follows: 120 minutes (43.2%), 30 minutes (30.4%), 60 minutes (21.6%), above 120 minutes (21.6%), other duration (9.6%) and less than 30 minutes (2.4%). Among the professionals who selected "other duration", seven out of twelve reported that it was based on patient characteristics ( Table 1 ).

The clinical parameters measured during SBTs included pulse oximetry saturation (92.8%), respiratory effort (89.9%), respiratory rate (86.2%), heart rate (82.6%), level of consciousness (82.6%), tidal volume (74.6%), the SpO_2_/FiO_2_ ratio (43.5%) and capnography (41.3%). For the second evaluation after the failure of the first SBT, the reported times for a new SBT were "after 24 hours" (69.3%), "after 48 hours" (17.5%), "after 12 hours" (10.2%) and "others" (3.6%).

### Methods of evaluating the ability to achieve spontaneous ventilation and its relationship with pediatric intensive care unit characteristics

The distributions of the three types of evaluations did not differ between university-affiliated hospitals and non-university-affiliated hospitals (p = 0.7), between general hospitals and pediatric hospitals (p = 0.6), or among different types of pediatric ICUs. The highest proportion of SBT use (55.6%) was recorded in the subgroup of university-affiliated pediatric hospitals. However, owing to segmentation and the small sample size, this difference was not statistically significant (calculated power: 68%). The only feature that behaved as a good discriminator was RT availability, dichotomized as an RT on duty for 12 - 24 hours *versus* all others (p = 0.006).

**Figure 2:**
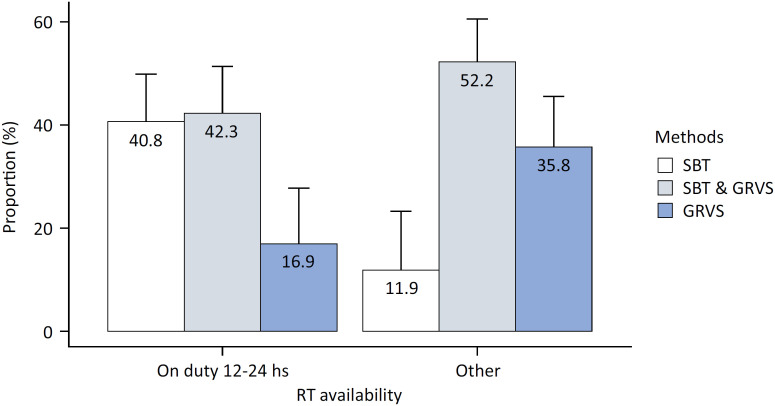
Distribution of methods according to the availability of respiratory therapists.

The distribution of methods between units with or without MV liberation protocols was 51.1% *versus* 14.3% for an SBT only, 14.9% *versus* 31.9% for the GRVS only, and 34.0% *versus* 53.8% for an SBT followed by the GRVS, respectively (p = 0.0001). These results did not differ between pediatric and general hospitals. The presence of an on-duty RT was associated with an OR of 5.1 (95%CI 2.1 - 12.5) for choosing an SBT as the main method. This result remained consistent after adjusting for pediatric *versus* general hospital status (OR 4.5; 95%CI 1.9 - 11.1) and university affiliation (OR 5.0; 95%CI 2.1 -12.5) ( Figure 2 ).

RT - respiratory therapist; SBT - spontaneous breathing trial; GRVS - gradual reduction of ventilatory support).

### Use of postextubation noninvasive ventilation

Preestablished criteria for the indication of noninvasive respiratory support (NRS) were reported to be absent in 40.4% of the patients. The most commonly prescribed rescue therapy was NIV with bilevel positive airway pressure (BiPAP) (43.5%), followed by "at the discretion of the responsible team" (42.8%) and CPAP (4%). No one reported using HFNC as the most common support method.

## DISCUSSION

This study is the first to examine the practices used during the process of liberation from invasive MV in the Ibero-American pediatric ICU setting. Only 34.1% (47/138) of the units used a standardized protocol. Nearly half of the units performed the GRVS followed by an SBT, whereas one-third of the units did not use an SBT prior to extubation. The most commonly employed SBT involved PS for 60 - 120 minutes. However, fewer than half of the units with a standardized protocol include criteria for the use of NRS postextubation. The only common characteristic among pediatric ICUs that used a standardized protocol and employed an SBT was the availability of an RT for at least 12 hours per day. The results of this survey highlight the need to standardize clinical practice for MV liberation in pediatric ICUs.

Five randomized clinical trials^( 13 - 17 )^ and three quality improvement projects^( 18 - 20 )^ support the use of MV liberation protocols. Additionally, recently published guidelines^( 12 , 21 )^ recommend adopting a bundle of measures to prepare for extubation or an ERT, aiming to reduce both the duration of MV and the risk of extubation failure. In our study, slightly more than 30% (47/138) of the pediatric ICUs used protocols, a percentage higher than that of 22% reported among 65 pediatric ICUs from 19 European countries,^( 22 )^ but lower than that of 46.8% reported in a survey among 380 international pediatric ICUs,^( 10 )^ and that of 57.5% reported in 146 pediatric ICUs in Brazil.^( 11 )^ The use of a standardized ERT over clinical judgment presents a valuable opportunity to enhance the care of children receiving invasive MV, and the first pediatric clinical practice guidelines on invasive MV liberation should serve as a supportive tool for implementing ERTs.

The results of this survey revealed high heterogeneity among Ibero-American pediatric ICUs. Fewer than one-third of the ICUs were affiliated with university hospitals, and only one-third were exclusively pediatric hospitals. The vast majority were classified as medical-surgical ICUs, with a low proportion being exclusively cardiovascular pediatric ICUs. Neither university affiliation, pediatric hospital affiliation, nor pediatric ICU type was associated with the use of standardized protocols for invasive MV liberation. No significant differences were observed when the cardiovascular pediatric ICUs were separated, considering that the effect of positive pressure may condition the liberation process on the basis of the underlying pathophysiology. The implementation of standardized protocols implies greater involvement of nurses and RTs in the weaning process, including the assessment of sedation.^( 23 )^ In our survey, the most common characteristic among pediatric ICUs with a standardized protocol was the presence of an RT for at least 12 hours per day. These findings contribute to existing evidence emphasizing the crucial role played by RTs and underscore the importance of multidisciplinary collaboration.^( 17 - 20 )^ Future multicenter trials should aim to demonstrate how adhering to clinical practice guidelines affects outcomes.

The international guidelines recommend the use of an SBT as part of the standardized liberation protocol. Surprisingly, almost one-third of the surveyed pediatric ICUs do not routinely incorporate SBT into their practices, despite other studies indicating more frequent SBT use in Latin America than in other geographical areas.^( 10 )^ Previously published studies conducted in Latin America may have already provided sufficient evidence for the widespread adoption of SBTs, including among postcardiovascular surgery patients.^( 13 , 24 - 26 )^ There is great variability in support methods used during SBTs, with half of the pediatric ICUs reporting the use of PS ranging from 5 - 7cmH_2_O and 30% reporting the use of a T-tube. Interestingly, the latter was chosen as the method of liberation from MV^( 13 , 24 )^ despite not being included in the international guidelines.^( 12 )^

The duration of SBTs varies from 10 to 120 minutes, likely due to the absence of comparative trials in pediatrics.^( 27 - 29 )^ In our survey, most pediatric ICUs performed SBTs for at least 30 minutes, with potentially longer trials for patients at a higher risk of extubation failure. The most frequently employed duration for SBT falls between 60 and 120 minutes. This is an area where guidelines lack evidence to support the duration of SBTs, and 60 - 120 minute tests are recommended for high-risk patients.^( 12 )^ Further clinical trials in this regard would be beneficial.

There are limited data on the benefit of postextubation NRS in preventing extubation failure. Noninvasive respiratory support treatment may prolong pediatric ICU and hospital stays, and there is no information in the pediatric literature concerning the use of preventive or rescue NRS. Forty percent of the respondents reported using preestablished criteria to support extubation with NIV or HFNC without being able to determine whether one type of support was superior to the other. An analysis of the postextubation NRS (FIRST-ABC trial) study^( 30 )^ concluded that HFNC, compared with CPAP, as postextubation support did not meet the noninferiority criterion for MV liberation. This study included patients under one year of age, and the international guidelines recommend CPAP over HFNC as for postextubation NRS in this age group.^( 12 )^

Our study has several limitations. First, the response rate was 48.9%, which reflects a lack of information from half of the Ibero-American pediatric ICUs. However, this response rate is consistent (44.1 - 52.3%) with that reported for other pediatric surveys that address the same topic.^( 9 - 11 )^ A higher response rate of 64% was only achieved in the European survey.^( 22 )^ Second, a common limitation shared with all surveys is that the data obtained rely on self-reports without data verification systems, resulting in estimates of the results in clinical practice. Finally, the heterogeneity of the sample, ranging from highly complex pediatric ICUs to those with limited resources, reflects practices that vary significantly. Nonetheless, we believe that the questionnaire provided a reliable assessment of the practices in each pediatric ICU, given that the completion of the survey was performed by a practitioner with enough knowledge of the liberation process.

## CONCLUSION

A large proportion of Ibero-American pediatric intensive care units have not yet adopted extubation readiness testing, including spontaneous breathing trials, into their practice. These findings present an opportunity to optimize the mechanical ventilation liberation process by implementing standardized protocols conducted by a multiprofessional team and supported by new clinical practice guidelines. The presence of a respiratory therapist was associated with increased adherence to guidelines, suggesting a modifiable factor that could enhance patient outcomes during liberation from mechanical ventilation.

## SUPPLEMENTAR MATERIAL




