# ERRATUM

**DOI:** 10.62675/2965-2774.20240066-enerr

**Published:** 2024-11-11

In the article **Clinical practices related to liberation from mechanical ventilation in Latin American pediatric intensive care units: survey of the *Sociedad Latino-Americana de Cuidados Intensivos Pediátricos* Mechanical Ventilation Liberation Group**, with DOI number: 10.62675/2965-2774.20240066-en, published in the journal Critical Care Science, 2024;36:e20240066en, on page title, affiliation number 3:

**Where it read:**

^3^ Department of Pediatrics, Universidade Federal de São Paulo - São Paulo (SP), Brazil.

**Read:**

^3^ Department of Pediatrics, Division of Pediatric Critical Care, Faculdade de Medicina, Universidade de São Paulo - São Paulo (SP), Brazil.

