# Supplementary material for: Clinical practices related to liberation from mechanical ventilation in Latin American pediatric intensive care units: survey of the Sociedad Latino-Americana de Cuidados Intensivos Pediátricos Mechanical Ventilation Liberation Group
Source: Crit Care Sci. 2024 Sep 5;36:e20240066en. doi: 10.62675/2965-2774.20240066-en (PMC11463992; doi:10.62675/2965-2774.20240066-en)
Supplement: Supplementary file 1 [file 2965-2774-ccsci-36-e20240066en-suppl01.pdf]

Critical Care Science
https://doi.org/10.62675/2965-2774.20240066-en

SUPPLEMENTARY MATERIAL

Clinical practices related to liberation from mechanical ventilation in Latin American pediatric intensive care units: survey of the *Sociedad Latino-Americana de Cuidados Intensivos Pediátricos* Mechanical Ventilation Liberation Group

Alejandra Retta[1], Analía Fernández[2], Ezequiel Monteverde[1], Cintia Johnston[3], Andrés Castillo-Moya[4], Silvio Torres[5], Jesus Dominguez-Rojas[6], Matias G. Herrera[7], Vlademir Aguilera-Avendaño[8], Yúrika López-Alarcón[9], Davi Pascual Rojas Flores[10], Manuel Eduardo Munaico-Abanto[11], Júlia Acuña[12], Rosa León[13], Carla Ferreira[14], Gabriela Sequeira[15], Cristina Camilo[16], Mauricio Yunge[17], Yolanda López Fernández[18]

Appendix 1 - Questionnaire

**1.**	**Profession of the respondent**
☐	Physician
☐	Respiratory therapist/Kinesiologist
☐	Nurse
**2.**	**Position in the unit (role)**
☐	Heads, directors
☐	Coordinator
☐	Staff
☐	Other (please specify) ____________
**3.**	**Health system where you work and are responding to this survey**
☐	Public health system institution
☐	Private health system institution
☐	Other (please specify) ____________
**4.**	**Type of hospital where the intensive care unit is located:**
☐	General hospital
☐	Pediatric hospital
☐	University hospital
☐	Other (please specify) ____________
**5.**	**Number of annual discharges from the pediatric intensive care unit as of December 2019: ____**
**6.**	**Type of intensive care unit**
☐	Medical
☐	Surgical
☐	Medical surgical
☐	Cardiovascular surgery
☐	All of the above
**7.**	**What is the age range of patients admitted to your unit?**
• Lower limit:	
☐	Admits patients from 7 days of life
☐	From 14 days of life
☐	From 30 days of life

Continue...

This is an open access article under the CC BY license (https://creativecommons.org/licenses/by/4.0/).

...continuation

• Upper limit:	
☐	Admits patients up to 14 years of age
☐	Up to 16 years of age
☐	Up to 18 years of age
**8.**	**Does your unit have a respiratory therapist**
☐	Attention by call or interconsultation
☐	Coverage of the ward 8 hours
☐	On-call 12 hours
☐	24-hour on-call
☐	No respiratory therapist
**9.**	**What is the method most frequently used to assessing extubation readiness?**
☐	Gradual reduction of ventilatory support, synchronized intermittent mandatory ventilation, pressure support reduction, etc.
☐	Spontaneous breathing trial (T- tube, continuous positive airway pressure, etc.)
☐	Gradual reduction of ventilatory support plus spontaneous breathing trial
☐	Not evaluated
**10.**	**If you selected gradual reduction of ventilatory support in the previous question, which of the following methods do you use?**
☐	Pressure support ventilation with guarantee volume
☐	Gradual reduction of pressure support ventilation
☐	Synchronized intermittent mandatory ventilation with gradual reduction of respiratory rate
☐	Other
**11.**	**If the previous answer includes spontaneous breathing trial, which of the following methods do you use?**
☐	Pressure support $5cmH_20$ + positive end-expiratory pressure $5cmH_20$
☐	Pressure support adjusted to endotracheal tube (ET) diameter + positive end-expiratory pressure $5cmH_20$
☐	Continuous positive airway pressure $5cmH_20$
☐	T-tube
**12.**	**In the case of using pressure support ventilation- positive end-expiratory pressure with other pressure support values, select the range of use**
☐	$< 5cmH_20$
☐	Between 5 - $7cmH_20$
☐	$> 7cmH_20$
☐	Depends on the case
**13.**	**Does your unit have a written liberation mechanical ventilation protocol?**
☐	Yes
☐	No
**14.**	**If yes, which of the following does it contain? Check all that apply**
☐	Established criteria for daily assessment of the possibility of mechanical ventilation liberation
☐	Sedoanalgesia protocol according to daily ventilatory support goals
☐	Standardized assessment test for extubation (including disconnection in patients with tracheostomy)
☐	Established criteria for failure of extubation
☐	Checklist for re-evaluation of causes of failure of the extubation readiness test
☐	Pre-established criteria for indication of noninvasive ventilation/high flow nasal cannula to prevent extubation failure

Continue...

...continuation

**15.**	**What is the duration of the liberation mechanical ventilation process?**
☐	< 30 minutes
☐	30 minutes
☐	1 hour
☐	2 hours
☐	> 2 hours
☐	Other (please specify) ___________________
**16.**	**What parameters do you monitor during the extubation readiness test?**
☐	Heart rate
☐	Respiratory rate
☐	Respiratory effort
☐	Exhaled CO_2
☐	SpO_2
☐	SpO_2/FiO_2 ratio
☐	Level of consciousness
☐	Tidal volume
☐	Other (please specify) ___________________
**17.**	**Do you perform any of the following tests?**
☐	Presence of cough pattern
☐	Swallowing
☐	Cuff leak test
☐	Evaluation of muscle strength
**18.**	**If the patient fails the extubation readiness test, how long is another attempt made?**
☐	The same day
☐	After 12 hours
☐	After 24 hours
☐	After 48 hours
**19.**	**How is successful weaning from mechanical ventilation defined in your workplace?**
☐	Patient not requiring reintubation within 24 hours
☐	Within 48 hours
☐	Within 72 hours
☐	Patient not requiring reintubation or noninvasive ventilation support within 24 hours
☐	Within 48 hours
☐	Within 72 hours
**20.**	**Do you consider extubation failure when the patient?**
☐	Is reintubated
☐	Receives noninvasive ventilation
☐	Receives high flow nasal cannula
**21.**	**Does your department use cuffed endotracheal tubes?**
☐	Yes
☐	No

Continue...

...continuation

**22.**	**If yes, is there a cuff pressure control protocol?**
☐	Yes
☐	No
**23.**	**Indicate your routine practice regarding prophylaxis of upper airway obstruction with corticosteroids**
☐	Administer corticosteroids before extubation and continue after extubation in all patients
☐	Prophylactic corticosteroids before extubation to patients at risk (previous extubation failure, laryngeal airway abnormalities, difficult intubation, etc)
☐	Do not give corticosteroids
☐	I do not know how to report
☐	Post-extubation nebulisation with epinephrine
**24.**	**When noninvasive support is indicated as a rescue to avoid reintubation, what is the established support in your pediatric intensive care unit?**
☐	High flow nasal cannula
☐	Continuous positive airway pressure
☐	Bi-level positive airway pressure
☐	Decision of attending physician
**25.**	**In your opinion, experience and knowledge, what is the term that should be used in a standardized way to refer to the process of weaning from invasive mechanical ventilation?**
☐	Weaning
☐	Withdrawal
☐	Liberation
☐	Other (please specify) ________________

Appendix 2 - Definitions, terms and nomenclature

**1.**	**Liberation from mechanical ventilation (LMV):** the process enabling the transition from invasive mechanical ventilation (MV) to spontaneous breathing and removal of the endotracheal tube (ETT).
**2.**	**Gradual reduction of ventilatory support (GRVS):** phase describing the transition from a controlled respiratory mode to other modes assisting the patient's inspiratory effort. This occurs due to patient improvement and/or stabilization.
**3.**	**Spontaneous breathing trial (SBT):** objective assessment of the patient's ability to independently maintain minute ventilation and adequate gas exchange without excessive respiratory effort using minimal pressure support (PS) or positive end-expiratory pressure (PEEP)/continuous positive airway pressure (CPAP).
**4.**	**Extubation readiness test (ERT):** bundle of elements used to assess the patient's eligibility for liberation from invasive mechanical ventilation. In addition to the SBT, ERT may include assessment of sedation level, adequacy of neurologic control of the airway (ie, cough and gag), likelihood of post-extubation upper airway obstruction, assessment of respiratory muscle strength, magnitude of airway secretions,hemodynamic status, and a plan for post-extubation respiratory support.
**5.**	**Extubation failure:** need for reintubation within 48 hours of extubation, excluding use for temporary procedures.
**6.**	**Noninvasive ventilatory support (NRS):** Refers to all respiratory supports without the need for an artificial airway: HFNC, noninvasive ventilation (NIV), CPAP, excluding conventional oxygen therapy.
**7.**	**High-flow nasal cannula (HFNC):** flow that is delivered through a heated humidified nasal cannula circuit and interface at a flow rate which is > 1L/kg/minute for patients up to 10kg; and > 10L/minute for patients above 10kg.
**8.**	**Continuous positive airway pressure:** CPAP that can be delivered via ETT, tracheostomy, or non-invasive interface (e.g., nasal mask, nasal cannula, full-face mask, or helmet).
**9.**	**Noninvasive ventilation:** ventilatory mode delivering positive pressure at different levels without the need for an artificial airway, e.g., nasal mask, nasal cannula, full-face mask, or helmet. For example, bi-level positive airway pressure (BiPAP).
**10.**	**Respiratory therapist (RT):** in Latin American UCIs the respiratory therapist is a physiotherapist who functions as a critical care practitioner, taking responsibility for managing patients with acute respiratory conditions or those requiring respiratory support in critical care settings. The outlined responsibilities include respiratory assessment, ventilator management, airway management, oxygen therapy, respiratory rehabilitation, and continuous monitoring, in collaboration with the whole ICU team.
**11.**	**Cuff:** the balloon of the endotracheal tube that seals the trachea to prevent aspiration of contents from the pharynx.
