# Supplementary material for: Clinical practices related to liberation from mechanical ventilation in Latin American pediatric intensive care units: survey of the Sociedad Latino-Americana de Cuidados Intensivos Pediátricos Mechanical Ventilation Liberation Group
Source: Crit Care Sci. 2024 Sep 5;36:e20240066en. doi: 10.62675/2965-2774.20240066-en (PMC11463992; doi:10.62675/2965-2774.20240066-en)
Supplement: Translation

# Translation: Práticas clínicas de desmame da ventilação mecânica em unidades de terapia intensiva pediátrica da América Latina: inquérito do Grupo de Liberação da Ventilação Mecânica da *Sociedad Latino-Americana de Cuidados Intensivos Pediátricos*

**DOI:** 10.62675/2965-2774.20240066-pt

**Authors:** Alejandra Retta, Analía Fernández, Ezequiel Monteverde, Cintia Johnston, Andrés Castillo-Moya, Silvio Torres, Jesus Dominguez-Rojas, Matias G. Herrera, Vlademir Aguilera-Avendaño, Yúrika López-Alarcón, Davi Pascual Rojas Flores, Manuel Eduardo Munaico-Abanto, Júlia Acuña, Rosa León, Carla Ferreira, Gabriela Sequeira, Cristina Camilo, Mauricio Yunge, Yolanda López Fernández

**Affiliations:** 1 Buenos Aires Argentina Unidade de Terapia Intensiva, Hospital General de Niños Ricardo Gutiérrez - Buenos Aires, Argentina.; 2 Buenos Aires Argentina Unidade de Terapia Intensiva, Hospital General de Agudos Carlos G. Durand - Buenos Aires, Argentina.; 3 São Paulo SP Brasil Departamento de Pediatria, Universidade Federal de São Paulo - São Paulo (SP), Brasil.; 4 Santiago Chile Unidade de Terapia Intensiva, Pontificia Universidad Católica de Chile - Santiago, Chile.; 5 Pilar Argentina Hospital Universitario Austral - Pilar, Argentina.; 6 Lima Peru Departamento de Pediatria, National Hospital Edgardo Rebagliati Martins - Lima, Peru.; 7 Buenos Aires Argentina Intensive Care Unit, Hospital de Pediatría Prof. Dr. Juan P. Garrahan - Buenos Aires, Argentina.; 8 La Paz Bolívia Unidade de Terapia Intensiva, Hospital del Niño Dr. Ovidio Aliaga Uría - La Paz, Bolívia.; 9 Medellin Colômbia Unidade de Terapia Intensiva, Hospital General de Medellín Luz Castro de Gutiérrez - Medellin, Colômbia.; 10 Chihuahua México Unidade de Terapia Intensiva, Hospital General Regional nº 1, Instituto Mexicano del Seguro Social - Chihuahua, México.; 11 Lima Peru Unidade de Terapia Intensiva, National Hospital Edgardo Rebagliati Martins - Lima, Peru.; 12 Asunción Paraguay Unidade de Terapia Intensiva, Instituto de Medicina Tropical - Asunción, Paraguay.; 13 Lima Peru Instituto Nacional de Salud del Niño - Lima, Peru.; 14 Assunção Brazil Unidade de Terapia Intensiva, Hospital Universitário San Lorenzo - Assunção, Paraguai.; 15 Montevidéu Uruguai Centro Hospitalario Pereira Rossell - Montevidéu, Uruguai.; 16 Lisboa Portugal Hospital de Santa Maria - Lisboa, Portugal.; 17 Las Condes Chile Unidade de Terapia Intensiva, Clínica Las Condes - Las Condes, Chile.; 18 Barakaldo Espanha Unidade de Terapia Intensiva, Hospital Universitário Cruces - Barakaldo, Espanha.

**Keywords:** Respiração artificial, Unidades de terapia intensiva pediátrica, Extubação, Ventilação não invasiva, Inquéritos e questionários

## Abstract

**Objetivo:**

Abordar a prática atual de desmame da ventilação mecânica invasiva de pacientes em unidades de terapia intensiva pediátrica, com foco no uso de protocolos, critérios, parâmetros e indicações padronizados para suporte respiratório não invasivo após a extubação.

**Métodos:**

Realizou-se uma busca eletrônica de novembro de 2021 a maio de 2022 em unidades de terapia intensiva pediátrica ibero-americanas. Participaram médicos e terapeutas respiratórios, com um único representante de cada unidade de terapia intensiva pediátrica incluída. Não houve intervenções.

**Resultados:**

A taxa de resposta foi de 48,9% (138/282), representando 10 países ibero-americanos. Em apenas 34,1% (47/138) das unidades de terapia intensiva pediátrica havia protocolos escritos de desmame de ventilação mecânica invasiva, e seu uso estava associado à presença de terapeutas respiratórios (RC 3,85; IC95% 1,79 - 8,33; p = 0,0008). O método mais comum de desmame envolveu uma redução gradual do suporte ventilatório mais um teste de respiração espontânea (47,1%). A duração média da tentativa de respiração espontânea foi de 60 a 120 minutos em 64,8% das respostas. A presença de um terapeuta respiratório na unidade de terapia intensiva pediátrica foi a única variável associada ao uso de um teste de respiração espontânea como o principal método de desmame da ventilação mecânica invasiva (RC 5,1; IC95% 2,1 - 12,5). Os protocolos de suporte respiratório não invasivo não foram usados com frequência após a extubação (40,4%). Quase metade dos respondentes (43,5%) relatou preferência pelo uso de pressão positiva de dois níveis nas vias aéreas como modo de ventilação não invasiva após a extubação.

**Conclusão:**

Uma grande proporção de unidades de terapia intensiva pediátrica ibero-americanas não possui protocolos de desmame. Nosso estudo destaca uma variabilidade substancial nas práticas de prontidão para extubação, ressaltando a necessidade de padronização desse processo. No entanto, a presença de um terapeuta respiratório foi associada a uma maior adesão às diretrizes.

## INTRODUÇÃO

Entre 35,7 e 55% dos pacientes internados em unidades de terapia intensiva (UTIs) pediátricas recebem ventilação mecânica (VM) invasiva.^( 1 - 4 )^Mesmo no contexto de vários processos patológicos críticos, o desmame precoce do suporte respiratório invasivo continua sendo objetivo prioritário para evitar riscos, complicações e aumento dos custos de saúde associados à VM invasiva prolongada.^( 5 - 8 )^

Apesar do amplo consenso sobre a minimização da duração da VM invasiva, a escassez de evidências sobre os métodos de desmame pediátrico contribui para uma variabilidade significativa na prática.^( 9 - 11 )^Recentemente, foram publicadas as primeiras *International Clinical Practice Guidelines for Pediatric Ventilator Liberation in Pediatrics* . Três recomendações principais enfatizam o uso de protocolos padronizados para identificar os pacientes que atendem aos critérios de desmame do ventilador e a aplicação de um conjunto de medidas para confirmar a prontidão para a extubação. Essas medidas devem incluir um teste de respiração espontânea (TRE).^( 12 )^

Embora a implementação dessas diretrizes possa contribuir para padronizar o desmame da VM em crianças, elas só começaram a ser divulgadas recentemente, e estudos anteriores revelaram variabilidade substancial nas práticas assistenciais.^( 1 - 5 , 7 - 11 )^

Elaboramos e realizamos um inquérito com o objetivo geral de examinar as práticas clínicas atuais relacionadas ao desmame da VM invasiva em UTIs pediátricas ibero-americanas. Os objetivos específicos incluíam: identificar o uso de protocolos padronizados; analisar os critérios e parâmetros usados durante o desmame da VM invasiva; e avaliar o uso de ventilação não invasiva (VNI) após a extubação. Os resultados do inquérito nos permitiram identificar áreas de melhoria e fornecer suporte para se desenvolverem estudos futuros que contribuam para a geração de evidências nessa importante área da medicina intensiva.

## MÉTODOS

### Desenho e população do estudo

O desenho do inquérito baseou-se em um documento original já publicado^( 11 )^com tradução para o espanhol, posteriormente adaptado pelo Grupo de Liberação da Ventilação Mecânica do Comitê Respiratório da *Sociedad Latino-Americana de Cuidados Intensivos Pediátricos* (SLACIP). O inquérito, inicialmente publicado em espanhol e posteriormente traduzido para o português no outono de 2021, realizou-se por meio de reuniões virtuais e foi distribuído a todos os países ibero-americanos por endereços de *e-mail* fornecidos pelos coordenadores nacionais afiliados ao Grupo de Liberação da Ventilação Mecânica. Cada *e-mail* incluía uma carta-convite, e um *e-mail* recordatório foi enviado após 2 a 4 semanas. Ao concordar em participar, um representante de cada UTI pediátrica preencheu o questionário, especificando sua profissão e função. Utilizou-se a plataforma Google Form, exigindo que os participantes digitassem um código de acesso predefinido.

### Desenvolvimento do inquérito

O inquérito continha 25 perguntas relacionadas ao processo de desmame da VM (Apêndice 1 - Material suplementar) e coletou o seguinte: características demográficas das UTIs pediátricas participantes, incluindo o tipo de sistema de saúde, de hospital e UTI pediátrica, o número e a faixa etária dos pacientes admitidos em 2019 (pré-pandemia da doença pelo coronavírus 2019 [COVID-19]); informações sobre recursos humanos, incluindo a presença de um terapeuta respiratório (TR); informações sobre a prática clínica, incluindo a definição do processo, o uso de um protocolo padronizado, o uso de um teste de prontidão para extubação (TPE) ou pacote de medidas para avaliar a aptidão de um paciente de ser desmamado da VM invasiva, incluindo o tipo e a duração do TRE (Apêndice 2 - Material Suplementar), o uso de outros testes, como o e fuga de ar, o uso de corticosteroides antes da extubação, os critérios de sucesso/falha no desmame e o prazo da realização de um novo TRE em caso de falha; e informações de suporte respiratório após a extubação.

Todos os inquéritos foram concluídos entre 2 de novembro de 2021 e 12 de maio de 2022. Excluíram-se os inquéritos que não continham dados demográficos e aqueles identificados como de alto risco para respostas duplicadas. As duplicatas foram determinadas pela comparação de respostas idênticas em relação à profissão da pessoa responsável por responder ao inquérito, tipo de hospital, número de altas anuais de cada unidade e financiador do centro de saúde. A isenção do Termo de Consentimento Livre e Esclarecido foi obtida pelo Comitê de Revisão Institucional do Hospital Universitário Austral de Buenos Aires (Argentina). Este estudo cumpriu os padrões éticos do comitê responsável pela experimentação humana, bem como a Declaração de Helsinque de 1975, com suas alterações mais recentes.

### Resultados e definições operacionais

O desfecho primário foi a análise das práticas atuais relacionadas ao desmame da VM em crianças no cenário ibero-americano de cuidados críticos. Essa análise se concentrou na descrição das características das UTIs pediátricas participantes, na disponibilidade de recursos humanos, na implementação do processo de desmame e na assistência respiratória após a extubação. Detalhes específicos sobre as definições operacionais podem ser encontrados no Apêndice 2 (Material Suplementar).

### Análise estatística

Procedeu-se a uma análise descritiva univariada. As variáveis categóricas são apresentadas como quantidade absoluta (n) e porcentagem, enquanto as variáveis numéricas são apresentadas como média e desvio-padrão (DP) ou mediana e intervalo interquartil (IIQ), conforme a distribuição observada. Nas comparações de resultados categóricos, utilizou-se o teste do qui-quadrado ou o teste exato de Fisher. No caso de variáveis numéricas, as medianas foram comparadas mediante análise de variância de Kruskal-Wallis. Valor de p < 0,05 indicou significância estatística. Todas as análises foram realizadas por meio do R (versão 4.2.1; 2018 The R Foundation for Statistical Computing Platform).

## RESULTADOS

Os convites de participação foram enviados a 282 UTIs pediátricas, e foram recebidos 138 inquéritos respondidos (taxa de resposta de 48,9%). Entre os inquéritos, 91,3% (126/138) foram respondidos por médicos e 8,7% (12/138) por TRs. Os chefes de departamento, diretores ou coordenadores de UTIs pediátricas responderam a 49,3% (68/138) dos inquéritos. A maioria dos respondentes era afiliada a hospitais públicos (73,9%, 102/38), e os hospitais pediátricos representavam quase um terço deles (31,2%, 43/138), sendo que 76,8% (106/138) não eram afiliados a uma universidade. Entre os hospitais afiliados a universidades, 28,1% (9/32) eram hospitais gerais. A taxa de alta da UTI pediátrica foi de 300 pacientes/ano (IIQ 142 - 432) ( Figura 1 e Tabela 1 ).

**Figura 1:** Altas anuais conforme o tipo de hospital.

GNU - geral não universitário; GU - geral universitário; PNU - pediátrico não universitário; PU - pediátrico universitário.

**Tabela 1:** Resumo das principais perguntas do inquérito e taxas de resposta

Perguntas do inquérito		Respostas %
Profissão do respondente (n = 138)		
	Médico	126/138 (91,7)
	Terapeuta respiratório	30/138 (8,3)
Sistema de saúde ao qual a instituição pertence (n = 138)		
	Público	102/138 (73,9)
	Privado	30/138 (21,7)
	Outro tipo	6/138 (4,3)
Tipos de instituição * (n = 138)		
	Universitária	32/138 (23,2)
	Geral	65/138 (47,1)
	Pediátrica	43/138 (31,2)
	Outros	7/138 (5,1)
Classificação do hospital por afiliação universitária (n = 32)		
	Geral	9/32 (28,1)
	Pediátrica	23/32 (71,9)
Tipos de UTI pediátrica (n = 138)		
	Médico-cirúrgica	95/138 (68,8)
	Médico-cirúrgica-cardiovascular	35/138 (25,4)
	Outros	8/138 (5,7)
Disponibilidade do terapeuta respiratório (n = 138)		
	Permanente	61/138 (44,2)
	Parcial (< 12 horas/dia)	58/138 (42)
	Indisponível	19/138 (13,8)
Método usado para avaliar a probabilidade de extubação (n = 138)		
	Somente TER *	37/138 (26,8)
	PS	26/37 (76,5)
	CPAP	2/37 (5,9)
	Tubo em T	13/37 (38,2)
	Somente RGSV	36/138 (26,1)
	Ambos (TRE + RGSV)	65/138 (47,1)
Protocolo escrito (n = 138)		
	Sim	47/138 (34,1)
	Não	91/138 (65,9)
Componentes do TPE * (n = 47)		
	Triagem diária	35/47 (74,5)
	Protocolo de sedoanalgesia	31/47 (66)
	Critérios para definir falha no TPE	31/47 (66)
	TRE	27/47 (57,4)
	Uso de VNI/CPAP após a extubação planejada	19/47 (40,4)
	Lista de verificação de critérios na falha	15/47 (31,9)
Duração do TRE * (n = 125)		
	< 30 minutos	3/125 (2,4)
	30 minutos	38/125 (30,4)
	60 - 120 minutos	81/125 (64,8)
	> 120 minutos	27/125 (21,6)
	Outra duração	12/125 (9,6)
Uso de TET com manguito (n = 138)		
	Sim	112/138 (81,2)
	Não	6/138 (4,3)
	Às vezes	20/138 (14,5)
Monitoramento da pressão do manguito do TET por protocolo (n = 112)		
	Sim	83/112 (74,1)
	Não	29/112 (25,9)
Corticosteroides para prevenir a obstrução das vias aéreas superiores (n = 125)		
	Todos os pacientes	49/125 (39,2)
	Pacientes com alto risco de obstrução das vias aéreas superiores	70/125 (56)
	Sem indicação	3/125 (2,4)
	Desconhecido	3/125 (2,4)

UTI - unidade de terapia intensiva; TRE - teste de respiração espontânea; PS - pressão de suporte; CPAP - pressão positiva contínua nas vias aéreas; RGSV - redução gradual do suporte ventilatório; TPE - teste de prontidão para extubação; VNI - ventilação não invasiva; TET - tubo endotraqueal.

* Permitiram-se múltiplas respostas.

Em termos de características das UTIs pediátricas, 68,8% (95/138) foram identificadas como UTIs médico-cirúrgicas e 25,4% (35/138) foram identificadas como UTIs médico-cirúrgicas-cardiovasculares. A idade mais jovem na admissão foi 30 dias em 64,5% (89/138) das unidades, enquanto em 29,7% (41/138) das UTIs pediátricas, a idade mínima foi de 7 dias. A idade mais avançada na admissão foi de 18 anos em 47,1% (65/138), 16 anos em 24,7% (34/138) e 14 anos em 28,3% (39/138) das UTIs pediátricas.

Com relação à disponibilidade de TRs em UTIs pediátricas, 44,2% (61/138) relataram dispor de 24 horas, 7,2% (10/138) relataram dispor de 12 horas e 10,9% (15/138) relataram dispor de 8 horas. Em 23,9% (33/138) das UTIs pediátricas, os TRs estavam acessíveis apenas para consulta interdisciplinar, enquanto 14% (19/138) das UTIs pediátricas não dispunham de TRs.

### Protocolos para desmame da ventilação mecânica invasiva: teste de prontidão para extubação

Apenas 47 das UTIs pediátricas incluídas (34,1%, 47/138) relataram ter um protocolo escrito para desmame da VM invasiva. Os elementos incluídos no TPE foram um protocolo de sedação e analgesia (66%), critérios para definir a falha no TPE (66%), um teste de ventilação espontânea padronizado (57,4%), critérios predefinidos de suporte após a extubação com VNI ou cânula nasal de alto fluxo (CNAF) (40,4%) e uma lista de verificação de falha de extubação (31,9%).

Outras medidas incluídas no TPE foram a presença de um reflexo de tosse (83,1%), deglutição (41,5%) e força muscular (26,2%). Ao todo, 66,9% dos profissionais relataram realizar testes de fuga como parte do TPE por meio de tubos endotraqueais (TETs) com manguitos desinflados. Com relação à utilização de TETs com balonete, condição necessária à realização do teste de fuga, a prevalência foi maior (91,5%) nas unidades com um TR em serviço do que naquelas sem (70,1%) um TR em serviço (p = 0,0027).

Quanto ao uso de corticosteroides, 56% dos profissionais relataram prescrever exclusivamente corticosteroides para pacientes de alto risco com tendência à obstrução das vias aéreas, enquanto 39,2% relataram prescrever exclusivamente corticosteroides para todos os pacientes. Além disso, 42,4% relataram administrar epinefrina nebulizada rotineiramente.

O TPE em UTIs pediátricas foi significativamente associado à presença de um TR em serviço e a protocolos de desmame de VM (p = 0,0008; razão de chance [RC] de 3,85; IC95% 1,79 - 8,33). Essa associação não foi afetada pelo tipo de hospital (RC de 3,70; IC95% 1,72 - 8,33) ou pelo tipo de UTI pediátrica (RC de 4,00; IC95% 1,85 - 8,33).

### Métodos para avaliar a capacidade de ventilar espontaneamente

O método mais comum para avaliar a prontidão para extubação foi a redução gradual do suporte ventilatório (RGSV) com TRE (47,1%). Em 26,8% das UTIs, foi relatado apenas TRE e, em 26,1%, apenas RGSV. Os respondentes podiam selecionar mais de uma opção do método e da duração. Entre os que relataram apenas TREs, 47,1% usaram ventilação com pressão de suporte (PS) em vez de pressão positiva expiratória final (PEEP - *positive end-expiratory pressure* ), 38,2% usaram um tubo em T, 29,4% usaram PS conforme o diâmetro do TET e 5,9% usaram pressão positiva contínua nas vias aéreas (CPAP - *continuous positive airway pressure* ). Entre as unidades que preferiram RGSV, 48,6% preferiram redução gradual da PS, 42,9% preferiram redução gradual da frequência respiratória durante a ventilação mandatória intermitente sincronizada (SIMV - synchronized intermittent mandatory ventilation), e 40% optaram pela ventilação com volume de suporte (VSV - *volume support ventilation* ) ( Tabela 2 ).

**Tabela 2:** Métodos de avaliação da capacidade de ventilação espontânea

Métodos		n (%)	IC95%
Avaliação da capacidade de respirar espontaneamente * (n = 138)			
	RGSV + TRE	65 (47,1)	38,6 - 55,8
	TRE	37 (26,8)	19,6 - 35,0
	RGSV	36 (26,1)	19,0 - 34,2
Métodos de TRE † (n = 37)			
	PSV acima da PEEP	16 (43,2)	27,1 - 60,5
	Tubo em T	13 (27,0)	20,2 - 52,5
	PSV conforme o diâmetro do TET	10 (27,0)	13,8 - 44,2
	CPAP	2 (5,4)	0,7 - 18,2
Métodos de RGSV † (n = 35) ‡			
	Redução gradual da PSV	17 (48,6)	30,4 - 64,5
	Redução gradual da frequência respiratória durante o uso do SIMV	15 (42,9)	25,5 - 59,2
	VSV	14 (40,0)	23,1 - 56,5
	Outros	1 (2,8)	0,7 - 14,5

IC95% - intervalo de confiança de 95%; RGSV - redução gradual do suporte ventilatório; TRE - teste de respiração espontânea; PSV - ventilação com pressão de suporte; PEEP - pressão expiratória final positiva; TET - tubo endotraqueal; CPAP - pressão positiva contínua nas vias aéreas; SIMV - ventilação mandatória intermitente sincronizada; VSV - ventilação com volume de suporte.

* Tamanho total da amostra;

† permitiram-se múltiplas respostas;

‡ o tamanho da amostra era 36, mas faltou uma resposta.

A duração relatada dos TREs foi a seguinte: 120 minutos (43,2%), 30 minutos (30,4%), 60 minutos (21,6%), acima de 120 minutos (21,6%), outra duração (9,6%) e menos de 30 minutos (2,4%). Entre os profissionais que selecionaram a opção "outra duração", 7 dos 12 relataram que ela se baseava nas características do paciente ( Tabela 1 ).

Os parâmetros clínicos medidos durante os TREs incluíram saturação por oximetria de pulso (92,8%), esforço respiratório (89,9%), frequência respiratória (86,2%), frequência cardíaca (82,6%), nível de consciência (82,6%), volume corrente (74,6%), relação entre saturação de oxigênio e fração inspirada de oxigênio (SpO_2_/FiO_2_) (43,5%) e capnografia (41,3%). Na segunda avaliação após a falha do primeiro TRE, os tempos relatados para um próximo TRE foram "após 24 horas" (69,3%), "após 48 horas" (17,5%), "após 12 horas" (10,2%) e "outros" (3,6%).

### Métodos de avaliação da capacidade de obter ventilação espontânea e sua relação com as características da unidade de terapia intensiva pediátrica

As distribuições dos três tipos de avaliações não diferiram entre hospitais universitários e hospitais não universitários (p = 0,7), entre hospitais gerais e hospitais pediátricos (p = 0,6) ou entre diferentes tipos de UTIs pediátricas. A maior proporção de uso de TRE (55,6%) foi registrada no subgrupo de hospitais pediátricos afiliados a universidades. Entretanto, devido à segmentação e ao pequeno tamanho da amostra, essa diferença não foi estatisticamente significativa (poder calculado: 68%). A única característica que se comportou como um bom discriminador foi a disponibilidade do TR, dicotomizada como um TR de plantão por 12 a 24 horas *versus* todos os outros (p = 0,006).

A distribuição de métodos entre unidades com ou sem protocolos de desmame de VM foi de 51,1% *versus* 14,3% no caso de apenas TRE, 14,9% *versus* 31,9% no caso de apenas RGSV e 34,0% *versus* 53,8% no caso de TRE seguido de RGSV, respectivamente (p = 0,0001). Esses resultados não diferiram entre hospitais pediátricos e gerais. A presença de um TR de plantão foi associada a uma RC de 5,1; IC95% 2,1 - 12,5 na escolha do TRE como método principal. Esse resultado permaneceu consistente após o ajuste do nível do hospital pediátrico *versus* hospital geral (RC de 4,5; IC95% 1,9 - 11,1) e afiliação à universidade (RC de 5,0; IC95% 2,1 - 12,5) ( Figura 2 ).

**Figura 2:** Distribuição dos métodos conforme a disponibilidade de terapeutas respiratórios.

TR - terapeuta respiratório; TRE - teste de respiração espontânea; RGSV - redução gradual do suporte ventilatório.

### Uso de ventilação não invasiva após a extubação

Foi relatada a ausência de critérios predefinidos para a indicação de suporte respiratório não invasivo (NRS - *noninvasive respiratory support* ) em 40,4% dos pacientes. A terapia de resgate mais comumente prescrita foi a VNI com pressão positiva de dois níveis nas vias aéreas (BiPAP - *bilevel positive airway pressure* ) (43,5%), seguida por "a critério da equipe responsável" (42,8%) e CPAP (4%). Não foi relatado o uso de CNAF como o método de suporte mais comum.

## DISCUSSÃO

Este estudo é o primeiro a examinar as práticas usadas durante o processo de desmame da VM invasiva no ambiente das UTIs pediátricas ibero-americanas. Apenas 34,1% (47/138) das UTIs utilizaram um protocolo padronizado. Quase metade das UTIs realizou RGSV seguido de TRE, enquanto um terço delas não utilizou TRE antes da extubação. O TRE mais comumente empregado envolveu PS por 60 a 120 minutos. Entretanto, menos da metade das UTIs com um protocolo padronizado inclui critérios para o uso de NRS após a extubação. A única característica comum entre as UTIs pediátricas que usavam um protocolo padronizado e empregavam TRE era dispor de um TR por pelo menos 12 horas por dia. Os resultados deste inquérito destacam a necessidade de padronizar a prática clínica do desmame de VM em UTIs pediátricas.

O uso de protocolos de desmame de VM é apoiado por cinco ensaios clínicos randomizados^( 13 - 17 )^e três projetos de melhoria de qualidade.^( 18 - 20 )^Além disso, diretrizes publicadas recentemente^( 12 , 21 )^recomendam a adoção de um conjunto de medidas de preparação para extubação ou TPE, com o objetivo de reduzir tanto a duração da VM quanto o risco de falha na extubação. Em nosso estudo, pouco mais de 30% (47/138) das UTIs pediátricas usavam protocolos, uma proporção maior do que a de 22% relatada em 65 UTIs pediátricas de 19 países europeus,^( 22 )^embora menor do que a de 46,8% relatada em um inquérito com 380 UTIs pediátricas internacionais,^( 10 )^e a de 57,5% relatada em 146 UTIs pediátricas no Brasil.^( 11 )^O uso de TPE padronizado em detrimento do julgamento clínico apresenta oportunidade valiosa para melhorar a assistência de crianças que recebem VM invasiva, e as primeiras diretrizes de prática clínica pediátrica sobre desmame de VM invasiva devem servir como uma ferramenta de apoio para a implementação de TPEs.

Os resultados desse inquérito revelaram alta heterogeneidade entre as UTIs pediátricas ibero-americanas. Menos de um terço das UTIs eram afiliadas a hospitais universitários e apenas um terço eram hospitais exclusivamente pediátricos. A maioria foi classificada como UTIs médico-cirúrgicas, com baixa taxa de UTIs pediátricas exclusivamente cardiovasculares. Nem a afiliação a uma universidade, nem a afiliação a um hospital pediátrico, nem o tipo de UTI pediátrica foram associados ao uso de protocolos padronizados para desmame invasivo de VM. Não foram observadas diferenças significativas quando as UTIs pediátricas cardiovasculares foram analisadas separadamente, considerando que o efeito da pressão positiva pode condicionar o processo de desmame conforme a fisiopatologia subjacente. A implementação de protocolos padronizados implica maior envolvimento de enfermeiros e TRs no processo de desmame, incluindo a avaliação da sedação.^( 23 )^Em nosso inquérito, a característica mais comum entre as UTIs pediátricas com um protocolo padronizado foi a presença de um TR por pelo menos 12 horas por dia. Esses achados contribuem para as evidências existentes que enfatizam o papel crucial desempenhado pelos TRs e ressaltam a importância da colaboração multidisciplinar.^( 17 - 20 )^Futuros estudos multicêntricos devem ter como objetivo demonstrar como a adesão às diretrizes de prática clínica afeta os desfechos.

As diretrizes internacionais recomendam o uso de TRE como parte do protocolo de desmame padronizado. Surpreendentemente, quase um terço das UTIs pediátricas que participaram do inquérito não incorporou o TRE em suas práticas de rotina, apesar de outros estudos indicarem o uso mais frequente do TRE na América Latina do que em outras áreas geográficas.^( 10 )^Estudos publicados anteriormente e realizados na América Latina podem já ter fornecido evidências suficientes para a adoção generalizada de TREs, inclusive entre pacientes em pós-operatório de cirurgia cardiovascular.^( 13 , 24 - 26 )^Há uma grande variabilidade nos métodos de suporte utilizados durante TREs, e metade das UTIs pediátricas relatou o uso de PS entre 5 e 7cmH_2_O, e 30% relataram o uso de um tubo em T. Curiosamente, este último foi escolhido como o método de desmame da VM,^( 13 , 24 )^apesar de não estar incluído nas diretrizes internacionais.^( 12 )^

A duração de TREs varia de 10 a 120 minutos, provavelmente devido à ausência de ensaios comparativos em pediatria.^( 27 - 29 )^Em nosso inquérito, a maioria das UTIs pediátricas realizou TREs por pelo menos 30 minutos, com ensaios potencialmente mais longos em pacientes com maior risco de falha na extubação. A duração mais frequentemente empregada em TREs fica entre 60 e 120 minutos. Essa é uma área em que as diretrizes carecem de evidências para respaldar a duração de TREs, e TREs de 60 a 120 minutos são recomendados para pacientes de alto risco.^( 12 )^Seria benéfico realizar mais ensaios clínicos a esse respeito.

Há dados limitados sobre o benefício do NRS após a extubação na prevenção da falha da extubação. O tratamento com NRS pode prolongar a permanência na UTI pediátrica e no hospital, e não há informações na literatura pediátrica sobre o uso de NRS preventivo ou de resgate. Dos respondentes, 40% relataram o uso de critérios predefinidos para apoiar a extubação com VNI ou CNAF sem poder determinar se um tipo de suporte era superior ao outro. Uma análise do estudo sobre NRS após a extubação (FIRST-ABC trial)^( 30 )^concluiu que a CNAF, em comparação com o CPAP, como suporte após a extubação, não atendeu ao critério de não inferioridade para desmame da VM. Esse estudo incluiu pacientes com menos de 1 ano de idade, e as diretrizes internacionais recomendam CPAP em vez de CNAF como NRS após a extubação nessa faixa etária.^( 12 )^

Nosso estudo tem várias limitações. Primeiramente, a taxa de resposta foi de 48,9%, o que reflete a falta de informações de metade das UTIs pediátricas ibero-americanas. No entanto, essa taxa de resposta é consistente (44,1 - 52,3%) com a relatada em outros inquéritos pediátricos que abordam o mesmo tópico.^( 9 - 11 )^Uma taxa de resposta mais alta, de 64%, só foi alcançada em um inquérito europeu.^( 22 )^Em segundo lugar, uma limitação comum compartilhada com todos os inquéritos é a de que os dados obtidos dependem de autorrelatos sem sistemas de verificação de dados, resultando em estimativas dos resultados na prática clínica. Por fim, a heterogeneidade da amostra, que vai desde UTIs pediátricas altamente complexas até aquelas com recursos limitados, reflete práticas que variam significativamente. No entanto, acreditamos que o questionário forneceu uma avaliação confiável das práticas em cada UTI pediátrica, uma vez que o preenchimento do inquérito foi realizado por um profissional com conhecimento suficiente do processo de desmame.

## CONCLUSÃO

Grande proporção de unidades de terapia intensiva pediátrica ibero-americanas ainda não adotou em sua prática o teste de prontidão para extubação, incluindo testes de respiração espontânea. Esses achados apresentam uma oportunidade de otimizar o processo de desmame da ventilação mecânica por meio da implementação de protocolos padronizados conduzidos por uma equipe multiprofissional e apoiados por novas diretrizes de prática clínica. A presença de um terapeuta respiratório foi associada à maior adesão às diretrizes, sugerindo um fator modificável que poderia melhorar os desfechos dos pacientes durante o desmame da ventilação mecânica.

## MATERIAL SUPLEMENTAR
