# Supplementary material for: ERRATUM
Source: Crit Care Sci. 2024 Nov 11;36:e20240066enerr. doi: 10.62675/2965-2774.20240066-enerr (PMC11812949; doi:10.62675/2965-2774.20240066-enerr)
Supplement: Related texts

# Correction: ERRATA

**DOI:** 10.62675/2965-2774.20240066-pterr

No artigo **Práticas clínicas de desmame da ventilação mecânica em unidades de terapia intensiva pediátrica da América Latina: inquérito do Grupo de Liberação da Ventilação Mecânica da** ***Sociedad Latino-Americana de Cuidados Intensivos Pediátricos***, com número de DOI: 10.62675/2965-2774.20240066-pt, publicado no periódico Critical Care Science, 2024;36:e20240066pt, na página de rosto, afiliação número 3:

**Onde se lia:**

^3^ Departamento de Pediatria, Universidade Federal de São Paulo - São Paulo (SP), Brasil.

**Leia-se:**

^3^ Departamento de Pediatria, Divisão de Cuidados Intensivos Pediátricos, Faculdade de Medicina, Universidade de São Paulo - São Paulo (SP), Brasil.
